# John De Haven White, M.D., D.D.S.

**Published:** 1896-02

**Authors:** 


### JOHN DE HAVEN WHITE, M.D., D.D.S.

THE announcement of the death of Dr. White in our last number was regretfully very brief for reasons then stated.

Dr. J. D. White was born in Lancaster, Pa., in 1815, and at the age of seven became an orphan and was bound out to a farmer. This was his humble beginning in a long struggle with the world. His educational advantages were very limited during this apprenticeship as a tiller of the soil.

In 1836 he was found in Philadelphia working at Jefferson College for a diploma in medicine. During his studies there he took private lessons in practical dentistry, the only method available at the time.

His practice, subsequent to graduation, was entirely confined to dentistry. Part of his early career was spent in Middletown, Pa., where be became the dentist of Senator Simon Cameron, and this laid the foundation for a friendship which lasted as long as the senator lived.

Dr. White began to lecture privately upon dental subjects in 1839, and continued these until the formation of the Philadelphia College of Dental Surgery, in 1852.

He was active in the formation of the Pennsylvania Association of Dental Surgeons, which held its first meeting in Peale's Museum in 1845; that society celebrated its fiftieth anniversary a few weeks ago at the Continental Hotel, erected upon that spot.

Dr. White was for ten years editor of the *Dental News Letter* and for six years editor of the *Dental Cosmos.*

He was a vigorous writer, expressing his opinions, apparently, without much anxiety as to results. The time required plain speaking, and Dr. White was not the man to measure words or modify sentences when he thought it necessary to be heard.

His practice for a considerable period in Philadelphia was very extensive, his clientèle covering the best families in that city. He received honorary degrees from various colleges, and was a member of most of the leading dental societies of the world.

The founder of the present house of S. S. White Dental Manufacturing Company studied under him, as did also Dr. Thomas W. Evans, of Paris. It is said that Napoleon III. urged Dr. White to join with Dr. Evans in forming in Paris a National Dental School. This offer Dr. White declined, as it would involve a large sacrifice of practice at home.

Those who were familiar with the subject of this sketch at the best periods of his career will remember him for his mental and physical vigor. His strong animal life seemed to fit him for an unlimited strain, marvellous to those who witnessed it. It seemed as though nothing could exhaust his vitality.

As a teacher he was vigorous and thorough, as this was understood at that early period in dental teaching. While there was a certain degree of hauteur in his manner he was generally popular with his students, as they recognized in him a reserve power always appreciated by the developing mind.

His work in paving the way for a higher professional life should be better appreciated to-day than when he was active on the stage.

The jealousies and frictions of that period are dead, and we can with truth assert that hard as his blows were, the iron will that gave them force was needed not alone in his own city, but in the profession at large. This way of dealing with things naturally caused opposition, but this is sometimes better than a dead level of weakness so often generated in a profession of fuller development. He was a true pioneer and blazed a path for others to follow. If the history of dentistry in the United States is ever written as it deserves, Dr. J. D. White will hold a prominent place in it, not so much as an original thinker, but as a powerful motive force in professional life.

The latter years of his life were spent in the quiet of the Masonic Home, Philadelphia, where he died in his eightieth year.

